# An Investigation into Helmet Use, Perceptions of Sports-Related Concussion, and Seeking Medical Care for Head Injury amongst Competitive Cyclists

**DOI:** 10.3390/ijerph19052861

**Published:** 2022-03-01

**Authors:** Jack Hardwicke, Brett Anthony Baxter, Tim Gamble, Howard Thomas Hurst

**Affiliations:** 1 Centre for Physical Activity and Life Sciences, University of Northampton, Northampton NN1 5PH, UK; brett.baxter@northampton.ac.uk; 2 The Department of Psychological Sciences, School of Psychology, Elizabeth Fry Building, University of Surrey, Guildford GU2 7XH, UK; t.gamble@surrey.ac.uk; 3 Centre for Applied Sport Physical Activity and Performance, University of Central Lancashire, Preston PR1 2HE, UK; hthurst@uclan.ac.uk

**Keywords:** sports-related concussion, helmets, competitive cycling, medical care seeking behaviors

## Abstract

The purpose of this study was to investigate competitive cyclists’ helmet use, perceptions of sports-related concussion (SRC), and medical-care-seeking behaviors. A mixed-method approach was used with qualitative and quantitative data presented. The study comprised of a cross-sectional analysis of 405 competitive cyclists who completed an online survey. Results indicated that most participants believed a bicycle helmet protects against SRC (79.5%) and considerable numbers of participants would not seek medical care for potential head injury in scenarios where this would be recommended. It was also discovered that marketing of concussion reduction technology influences cyclists’ helmet-purchasing behaviors. With the data presented, it is recommended that governing bodies in cycling need to develop educational resources to address gaps in knowledge regarding SRC amongst cyclists. We also suggest that more independent research on concussion reduction technologies in bicycle helmets is needed, with advertising supported by clear scientific evidence to avoid negatively influencing head injury management and reporting behaviors amongst cyclists.

## 1. Introduction

The health implications of sports-related concussion (SRC), a form of traumatic brain injury, have been an ongoing and increasing concern in sports medicine. US research suggests that 1.6–3.8 million SRCs are diagnosed annually [1] with as many as fifty percent of concussions going unreported [2]. International attention on SRC can largely be attributed to the potential long-term negative effects for brain health of both concussive and sub-concussive injuries [3,4,5,6]. 

Whilst much of the scientific and cultural focus of SRC has been on contact sports, particularly American Football [7] and Rugby [8], there have been concerns raised amongst cycle sports [9,10,11,12]. Bicycling, recreationally and competitively, is an increasingly popular activity across the world. For example, in the United Kingdom, British Cycling had 150,000 active members in 2019, representing a three-fold increase since 2012, and the largest recorded membership base since its establishment [13]. Importantly, bicycling has also been highlighted as one of the leading causes for mild traumatic brain injury requiring treatment in US emergency departments [14]. Recent research also estimates that head injuries (including SRC) make up 5–15% of injuries in road cycling [15] and between 5 and 13% in mountain biking [16,17,18]. Therefore, it is germane that cycle sports be included in research focused on SRC. 

In recent years, there have been concerns raised in road cycling disciplines regarding concussion incidence, policy, and attitudes amongst athletes [9]; however, research remains somewhat limited in this area [10,19,20,21]. In mountain biking and bicycle motocross (BMX), Hurst and colleagues [12,22,23] report that these athletes may be at risk of sub-concussive brain trauma due to the uneven terrain encountered. Hurst et al. [12] observed transient reduced executive functioning following only one day of downhill mountain biking. Other disciplines remain understudied, and the field is in its infancy irrespective of discipline. 

The focus of this paper is on competitive cyclists’ knowledge and perceptions of helmet use as they relate to SRC, an area currently not well investigated in the literature. This is of interest because helmet use in competitive cycling is now mandated by national and global governing bodies. For example, the Union Cycliste Internationale (UCI) mandated helmet use in all sanctioned events in 2003 [24]. This was largely due to research showing the use of bicycle helmets leading to an overall reduction in risks associated with head injury from cycling [25,26]. Indeed, helmet use is associated with reduced risk of structural head injury and death [27,28]; however, the research is less concrete when examining the role of bicycle helmets and protection from SRC [28,29].

There is limited biomechanical data on bicycle helmets protective capacities outside of industry regulation. Current safety standards are designed to reduce the risk of catastrophic injury or death by enforcing a lower limit on the linear acceleration metric in helmet impact testing [30,31]. However, the tests used to inform these standards do not reflect the biomechanical reality of bicycle crashes, where cyclists experience both linear and rotational kinematics [32,33]. This is important as research now highlights the role of rotational forces in producing mild traumatic brain injury [34]. Despite this, there are currently no safety standards for helmet evaluation that includes testing for rotational forces.

Additionally, the standard helmet drop height onto a flat anvil test, as used by the Consumer Product Safety Commission (CPSC), is 2 m, with a resulting impact velocity of 22.5 km·h^−1^. Whilst this may be representative of non-competitive cyclists, it is far slower than the 40.5 km·h^−1^ mean race velocities observed across bicycle races at the world tour professional level [35]. Another possible limitation of helmet testing standards is the combined mass of the drop test assembly, which is 5 kg excluding the helmet [36]. Taking even the heaviest helmets into consideration, the total mass is unlikely to exceed 6.5–7 kg. Given that the impact forces will be influenced by total body mass when an individual crashes, drop test velocity and equipment mass may potentially greatly underestimate impact forces riders experience during real world accidents.

In recent years, some bicycle helmet manufactures have developed technologies promoted as reducing the risk of concussion to cyclists. The two leading technologies in this area currently being marketed are the multi-directional impact protection system (MIPS), which has a slipcover inside the helmet allowing sliding between the head and helmet on impact, and WaveCel, which uses an angular impact mitigation (AIM) system with an inner lining to absorb accelerations during impact. There is currently a paucity of scientific evidence that either technology can reduce concussion injury risk and limited independent testing of the technologies exists. However, there have been a handful of studies assessing the effectiveness of these technologies in reducing rotational impact and brain injury risk [37,38,39]. DiGiacomo et al. [40] recently extended this inquiry into snow sport helmets with findings claiming that rotation-damping systems in snow sport helmets can reduce rotational head acceleration and the associated concussion risk. Considering the lack of official testing, research by Bland et al. [33] proposed a system to assess the ability of helmets to withstand rotational forces.

This small body of research on concussion reduction technologies is promising; however, there are significant limitations to the work at present. The two primary concerns with the research are that results were obtained under specific impact angles and velocities, potentially reducing the validity in real-world bicycle crashes, with all papers acknowledging results cannot be extrapolated to real-life scenarios. Second, the authors highlight the uncertainty of defining brain tolerance limits and the non-linear nature of brain injury risk [38]. This limitation is also highlighted by Fahlsted et al. [41] more generally on helmet testing that includes computational brain injury models, where they conclude this measure should not be used in helmet rating scales.

There appears to be no further evidence of the effectiveness of concussion reduction technologies and there is limited available data on the clinical outcomes for real-life patients. It should also be noted that the available research papers evaluating the effectiveness of these technologies have all reported conflicts of interest due to financial investments in the products being tested. Furthermore, there has been a recent lawsuit over the claims made for the protective capabilities of the WaveCel technologies regarding concussion risk and how this is communicated to the public [42]. 

Whilst there is some preliminary research on evaluating helmets for concussion protection, there is limited research on cyclists’ understanding of this and how concussion risk is communicated to the product users and athletes in cycling. The only previous study located was conducted by O’Reilly and colleagues [20], where the primary focus was on concussion knowledge and attitudes amongst adult cyclists. In their study, only 12.8% of the sample (*n* = 672) correctly reported that helmets were not able to prevent concussion. When this finding is coupled with the low reporting rates and dangerous attitudes toward concussion typical amongst competitive cyclists [10,43], an area in need of further research can be highlighted. In addressing this gap in knowledge, this research sought to establish competitive cyclists’ perceptions of helmet use, SRC and seeking of medical care for potential head injury.

## 2. Materials and Methods

### 2.1. Participants

Four hundred and five participants, all of whom declared they raced competitively, completed an anonymized online survey. The mean participant age was 43 ± 13 (range 18–80 years). The cohort was mixed-sex, with males (*n* = 347), females (*n* = 56), prefer not to say (*n* = 1), and other (*n* = 1). Competitive abilities included novice (*n* = 56), regional/club-level (*n* = 255), national (*n* = 69), and elite (*n* = 25), with a mean competitive experience of 14 ± 12 years. Though the sample was international, most respondents were from the USA and UK. Table 1 shows the location and frequency of respondents.

Survey respondents were from a heterogeneous range of disciplines. The frequency of responses by racing discipline are presented in Table 2.

### 2.2. Procedures

The survey was constructed amongst the research team on Microsoft Forms [44] and a pilot study was run on 15 competitive cyclists to gather feedback and assess validity before the final version was agreed by the research team. The survey was then disseminated via Twitter, Facebook, and specific online cycling forums between May and August 2021. The survey consisted of four sections. Section 1 determined demographic information along with primary racing disciplines, years of racing and experience level, primary country of racing, and whether they wear a helmet (or not) and why. Section 2 consisted of four questions to establish rider perceptions of helmet safety, with a possible ‘True’, ‘False’, or ‘I am unsure’ answer. Section 3 surveyed crash management behavior via 3 Likert scale questions, with responses being ‘Strongly Disagree’, ‘Disagree’, ‘Neutral’, ‘Agree’, or ‘Strongly Agree’. Finally, Section 4 was made up of 3 questions to determine helmet-purchasing behavior. These questions were a mix of ‘Yes’, ‘No’, or ‘Maybe’ questions, as well as multiple choice and open field questions.

### 2.3. Data Analysis

Data were analyzed using IBM SPSS Statistics for Windows, Version 26 [45]. Chi-square tests of independence were conducted to assess the influence of sex, age, discipline, and ability level on frequency of survey response rates. To examine the influence of age on survey response rates, three groups were created (18–30, 31–49, and 50+ years of age). If >20% of cells in the contingency tables had an expected count <5, the Fisher’s exact test was used. Where contingency tables were larger than 2 × 2, Fisher–Freeman–Halton tests were used. The McNemar test was conducted to assess within-subject differences in response to the question terminology used. Mann–Whitney U tests were conducted to assess between-sex differences for individual Likert scale responses, and Kruskal–Wallis tests were conducted to assess between-ability level differences for individual Likert scale responses. Post-hoc analyses were conducted using Tukey’s test to examine where differences between ability levels occurred. When examining the influence of sex on the frequency of survey response rates, participants that answered “other” and “prefer not to say” were removed from the analyses given the small sample size for each category (“other” *n* = 1; “prefer not to say” *n* = 1); therefore, the analyses were conducted on 403 participants. When examining the effect of question terminology, participants who answered “I am unsure” were removed to ensure that the data were dichotomous; therefore, data were analyzed from participants that answered “true” or “false” (*n* = 316). Statistical significance for all tests was accepted at *p* < 0.05.

The responses to the two open field questions “Why do you wear a helmet (or not)? Please give a brief summary in your own words” and “Do you have any comments on concussion reduction technology in bicycle helmets?” were coded and collated into key categories using conventional content analysis [46]. 

### 2.4. Ethics

Ethical approval was obtained at faculty level from The University of Winchester prior to the study commencing (reference number: HWB_REC_210329). A participant information sheet was provided as a preamble to the survey and formed the first page of the survey. Informed consent was implied if the participant continued with the survey. It was made clear to the participant that withdrawal was possible at any time during the survey by simply closing their web browser and their data would not be saved.

## 3. Results

### 3.1. Helmet Use

Most participants reported that they would wear a helmet in competitions, even if not compulsory (95.1%). There were no significant differences in this response between sexes or ability levels (*p* > 0.05). There was a significant difference between age groups (χ^2^ = 10.752; *p* = 0.004); 18–30 (“Yes” = 100.0%; “No” = 0.0%), 31–49 (“Yes” = 96.4%; “No” = 3.6%) and 50+ (“Yes” = 90.4%; “No” = 9.6%). Adults 50+ were less likely to wear a helmet if not compulsory than adults aged 18–30. There was a significant difference between disciplines (χ^2^ = 39.956; *p* < 0.001); audax/long-distance cyclists tended to be less likely to report wearing a helmet if not compulsory.

Analysis of open field responses to “Why do you wear a helmet (or not)? Please give a brief summary in your own words” found that safety and less injury severity were the primary reasons. Considerable numbers of participants (*n* = 38) reported specifically wearing a helmet to mitigate against concussion. Example responses included “To prevent more concussion” and “Reduces chance of concussion or serious head injury”. In response to the most important factors when purchasing a new helmet, participants rated fit, safety, and aerodynamics as the primary considerations.

### 3.2. Helmet Knowledge

Knowledge of helmets’ reducing risk of skull fractures was high amongst all respondents. However, only 20.5% correctly reported that a bicycle helmet does not protect against concussion. When asked about traumatic brain injury, 17.5% reported that a helmet does not offer protection. The McNemar test revealed a non-significant difference in participant responses between the wording of the question, i.e., “A bicycle helmet protects against concussion in the event of a crash” vs. “A bicycle helmet protects against traumatic brain injuries in the event of a crash” (*p* = 0.144).

Most respondents reported that they believed a helmet protects against concussion and traumatic brain injury. There were no significant differences in this response between age, sexes, or ability levels (*p* > 0.05). There were significant differences found amongst disciplines for knowledge of protection against concussion (χ^2^ = 30.681; *p* = 0.013); BMX and audax/long-distance cyclists tended to be more likely to report correctly that a helmet does not protect against concussion compared to other disciplines. There were also significant differences found amongst disciplines for knowledge of protection against traumatic brain injuries (χ^2^ = 29.787; *p* = 0.015); BMX and audax/long-distance cyclists tended to report correctly that a helmet does not protect against traumatic brain injuries compared to other disciplines. 

### 3.3. Helmet Damage and Seeking Medical Care

Responses to seeking medical care for potential head injury after three scenarios can be seen in Table 3. There were no significant differences between age, sex, and ability to seek medical care for a potential head injury for each three of the scenarios (*p* > 0.05). A significant difference was found between disciplines for seeking medical care for a potential head injury after a crash with a scuffed helmet (*p* = 0.001); post-hoc pairwise comparisons revealed that mountain biking cyclists were less likely to seek medical care for a potential head injury if the helmet was scuffed after a crash compared to cyclo-cross cyclists (*p* = 0.002) and triathlon and/or duathlon cyclists (*p* = 0.010). No significant differences were found between other disciplines (*p* > 0.05).

A significant difference was found between disciplines for seeking medical care for a potential head injury after a crash with a cracked helmet (*p* = 0.013); post-hoc pairwise comparisons revealed that mountain biking cyclists were less likely to seek medical care for a potential head injury if the helmet was cracked after a crash compared to cyclo-cross cyclists (*p* = 0.020). No significant differences were found between other disciplines (*p* > 0.05).

A significant difference was found between disciplines for seeking medical care for a potential head injury after a crash where the helmet was not damaged but there was contact with the floor (*p* = 0.001). Post-hoc pairwise comparisons revealed that BMX cyclists would not seek medical care for potential injury compared to cyclo-cross cyclists (*p* = 0.048). No significant differences were found between other disciplines (*p* > 0.05). If the helmet was cracked, most respondents reported they would replace it with a new one (“Agree” = 15.3%, “Strongly Agree” = 80.7%).

### 3.4. Concussion Reduction Technologies in Helmets

Respondents were asked “Has helmet marketing that advertises protection against concussion ever influenced your decision to buy the product? (e.g., MIPS, WaveCel)”. Responses were split between “Yes” (42.5%), “No” (43.2%), and “Maybe” (14.3%). There were no significant differences between sexes and ability levels (*p* > 0.05). Significant differences were found between disciplines (χ^2^ = 33.265; *p* = 0.006); audax/long-distance riders were less likely to be influenced by advertising than other disciplines. Significant differences were found between age groups (χ^2^ = 11.891; *p* = 0.018); the 18–30 age group was most influenced by marketing.

Analysis of open-field responses to “Do you have any comments on concussion reduction technology in bicycle helmets?” revealed that a considerable number (*n* = 57) of the participants were critical of the evidence supporting this technology. Example responses include: 

“Important, but needs publicly available scientific results and transparency, third-party evaluation”

“I don’t believe a helmet can stop your brain crashing into your skull in the event of a sudden incident.”

“Doesn’t work. 12.5 m/s is designed to protect those with existing brain injuries from further damage from a standing fall. MIPS only reduces the extra risk introduced by wearing a helmet, that would not be there otherwise.”

“Lack of consistent information and access to information about the technology”

“It’s unproven”

## 4. Discussion

Sports-related concussion (SRC) is an increasing medical concern in sport. As covered in our literature review, this concern is only recently being considered within cycle sports. However, it is estimated that SRC make up 5–15% of injuries in road cycling [15] and between 5–13% in mountain biking [16,17,18], with incidence data not currently available in other disciplines. Cycling has also been identified as one of the leading causes for mild traumatic brain injury in US emergency departments [14]. Previous research has highlighted that competitive cyclists would not disclose SRC in order to remain in competition [10,20,43]. 

With the increased cultural attention on SRC, we sought to understand what competitive cyclists’ perceptions of helmet use were in relation to SRC. This is of interest when considered in line with the literature on helmets not offering protection against concussion [28,29]. Moreover, the increase in helmet technologies being marketed as offering specific protection from concussion without strong scientific evidence, as problematized in our literature review. Our interest, and concern, is how competitive cyclists’ knowledge of helmet protection may influence their head injury management behaviors. 

The current study found that considerable numbers of competitive cyclists believe a helmet protects them from SRC. Our finding supports the work O’Reilly and colleagues [20] which showed that large numbers of cyclists also held this belief. This has potential to be highly problematic and contradicts with current scientific evidence and the biomechanics of brain injury. Previous research identifies competitive cyclists exhibiting harmful attitudes towards concussion and injury management, despite holding knowledge of the severity of the injury [10,20,43]. The work of Dahliquist and colleagues [47] also suggests that cycle sport athletes are more prone to competing whilst injured. We suggest that a false sense of security in SRC protection may contribute to cyclists not reporting head injuries, seeking medical care, or opting to continue in competition—i.e., the helmet “did its job” attitude.

Furthermore, people’s perception of safety can influence their engagement with risk. The theory of risk compensation posits that people will adjust behaviors in response to the perceived level of risk [48]; as such, the theory would predict people are more likely to engage in a risky activity if they feel more protected from such a risk. Gamble and Walker [49] found that helmet use significantly increased risk-taking and sensation-seeking behaviors in a laboratory task, when compared to a group wearing a baseball cap, which does not carry the safety connotations of a helmet. It is possible that cyclists who believe in the concussion reduction technology marketing in helmets may be more prone to engaging in risk. In terms of the data presented in the current study, this might also manifest through the numbers reporting they would not seek medical care after a crash, and previous recent research has found that competitive cyclists would continue in competition following a concussion [10,18,43].

Whilst most cyclists believed helmets could protect them from SRC, significant differences were revealed between age groups relating to compulsory helmet use, with the 50+ group reporting that they would be less inclined to wear a helmet in races if not compulsory. This may be a generational difference, as many of these riders may have previously raced in an era when helmets were not compulsory without sustaining SRC, whereas younger riders may not have had that experience.

The different terminology of “Concussion” or “Traumatic brain injury” did not have a significant effect on participant responses. Both terms represent the same injury and we tested for this as there is a body of research recognizing the impact of the different terms on athlete perception and appreciation of the severity of the injury [50,51,52]. However, in this study, competitive cyclists were not influenced by the different terminology. 

Encouragingly, the majority participants in the current study identified that they would seek medical care for a potential head injury if they were involved in a crash where the helmet was cracked, as detailed in Table 3. However, it is worth noting that considerable numbers reported “Neutral”, “Disagree”, and “Strongly disagree” to this (Table 3). This finding supports previous research where cyclists were found to under-report and not seek care for head injury in considerable numbers [10,20,43].

Further, when the helmet was only ‘scuffed’ or when the crash was high-impact, but the head did not contact the floor, considerable numbers of the participants reported they would not seek medical care for head injury. This is potentially problematic as, contrary to popular belief, impacts to other areas of the body can cause the biological responses leading to a concussion [53]. This apparent gap in cyclist’s knowledge may contribute to an underreporting of head injury and reduced rates of seeking medical care.

Given that a considerable number of respondents believed that helmets did protect against SRC, a scuffed helmet may be viewed as superficial damage and, given the believed protective benefits of helmets, unlikely to result in a head injury, resulting in them not seeking medical attention. Results also indicated that mountain bike cyclists were statistically more likely not to seek medical attention than riders from other disciplines. This may be linked to greater risk-taking behavior observed in this group, as previously reported by Frühauf et al. [54]. Such risk-taking attitudes may also result in mountain bike riders ‘trivialising’ head injuries more than those from other disciplines, in part contributed to by the extreme or dangerous nature of mountain biking compared to other disciplines. This trivialization of head injuries was also found by Clark et al. [18], who reported that 67.5% of surveyed mountain bike cyclists (*n* = 219) reported that they would continue riding with known concussive symptoms, while 29.2% reported they would continue riding with a broken helmet.

Results from the current study also indicated that a large number of our participants were influenced by helmet marketing that advertised for protection against concussion. This was expected, particularly considering the increased media and cultural attention to SRC in recent years. However, with the concerns raised over this technology in our literature review, we caution against the advertisement of these products for the influence it may have on cyclist perceptions and head injury reporting behaviors. Indeed, the qualitative analysis found some skepticism around these technologies amongst some of the participants. Older riders were less influenced by marketing than those aged between 18 and 30 years old. This finding is supported by Carpenter and Yoon [55] who found that older consumers did not engage well with information overload, whilst market research from marketing agency ‘Coming of Age’ [56] who specialize in advertising campaigns targeting older consumers reported those 50+ were less responsive to marketing claims and were more likely to consider purchases based on facts. We suggest that more rigorous and independent research is required before helmet manufactures can ethically use protection from concussion in advertising.

In line with current evidence, it is recommended that governing bodies and practitioners in the sport place efforts on increasing education around helmets and SRC, as well as awareness around SRC in cycle sports more broadly. This is something that previous research is currently lacking [9,10,43]. Furthermore, a recent scoping review from Scullion and Heron [57] on concussion guidelines in amateur sports in the UK found British Cycling to be one of only two governing bodies to not have published SRC guidelines available to the public. 

## 5. Conclusions and Recommendations

The current study found that considerable numbers of competitive cyclists believe that a bicycle helmet protects them from SRC, as well as a substantial amount not seeking medical care for head injury in cases where this would be recommended. Furthermore, it was found that an increase in new helmet technologies focused on reducing concussion is influencing many cyclists’ purchasing behaviors.

In light of these findings, it is recommended that clinicians, practitioners, and stakeholders in the sport ensure that SRC protocols are adhered too. To aid this, governing bodies need to implement more robust in-competition protocols for all levels of the sport, as well as develop educational resources to address apparent gaps in knowledge regarding SRC and the effectiveness or limitations of current helmet technologies.

Finally, we suggest that more independent research is needed on such concussion reduction technologies in bicycle helmets. The current market advertising without strong scientifically rigorous evidence has the potential to negatively influence head injury management and reporting behaviors amongst cyclists, as well as contribute to misunderstandings around the role of bicycle helmets and SRC.

## Figures and Tables

**Table 1:** Survey respondent frequency by geographic location.

Country	Survey Frequency Response (*n* = 405)
Australia	6
Brazil	1
Canada	14
France	1
Germany	3
India	1
Netherlands	2
New Zealand	1
Philippines	1
South Africa	2
Spain	6
Sweden	1
Switzerland	1
United Kingdom	242
United States	123

**Table 2:** Survey respondent frequency by bicycle racing discipline.

Discipline	Survey Frequency Response (*n* = 405)
Road cycling	138
Time trailing	60
Mountain biking	47
Downhill mountain biking	4
BMX	5
Cyclo-cross	20
Track cycling (including grass track)	9
Triathlon and/or duathlon	99
Audax/long distance	20
Other	3

**Table 3:** Frequency of Likert survey responses (*n* = 405).

	Frequency of Likert Survey Responses				
Statement	Strongly Disagree	Disagree	Neutral	Agree	Strongly Agree
“I would seek medical care for potential head injuries if I had been involved in a high impact crash, but my head (and helmet) did not contact the floor.”	49 (12.1%)	132 (32.6%)	128 (31.6%)	65 (16.0%)	31 (7.7%)
“I would seek medical care for potential head injuries if I had been involved in a crash where my helmet had been scuffed but not cracked.”	43 (10.6%)	149 (36.8%)	126 (31.1%)	59 (14.6%)	28 (6.9%)
“I would seek medical care for potential head injuries if I had been involved in a crash where my helmet had been cracked.”	9 (2.2%)	47 (11.6%)	87 (21.5%)	140 (34.6%)	122 (30.1%)

## Data Availability

All data underpinning this publication are openly available from the University of Northampton Research Explorer at: http://doi.org/10.24339/e5e993a0-276d-42ff-a061-0664329b6075 (accessed on 11 January 2022).
